# Living without DAT: Loss and compensation of the dopamine transporter gene in sauropsids (birds and reptiles)

**DOI:** 10.1038/srep14093

**Published:** 2015-09-14

**Authors:** P. V. Lovell, B. Kasimi, J. Carleton, T. A. Velho, C. V. Mello

**Affiliations:** 1 Department of Behavioral Neuroscience; Oregon Health & Science University; Portland, OR 97239-3098; USA; 2 Department of Biology; Portland State University; Portland, OR 97207-0751; USA; 3 Present address: Biology Program; University of Utah; Salt Lake City, UT 84112-9057; USA; 4 Present address: Institute; Federal University of Rio Grande do Norte, Natal, RN 59056-450; Brazil

## Abstract

The dopamine transporter (DAT) is a major regulator of synaptic dopamine (DA) availability. It plays key roles in motor control and motor learning, memory formation, and reward-seeking behavior, is a major target of cocaine and methamphetamines, and has been assumed to be conserved among vertebrates. We have found, however, that birds, crocodiles, and lizards lack the DAT gene. We also found that the unprecedented loss of this important gene is compensated for by the expression of the noradrenaline transporter (NAT) gene, and not the serotonin transporter genes, in dopaminergic cells, which explains the peculiar pharmacology of the DA reuptake activity previously noted in bird striatum. This unexpected pattern contrasts with that of ancestral vertebrates (e.g. fish) and mammals, where the NAT gene is selectively expressed in noradrenergic cells. DA circuits in birds/reptiles and mammals thus operate with an analogous reuptake mechanism exerted by different genes, bringing new insights into gene expression regulation in dopaminergic cells and the evolution of a key molecular player in reward and addiction pathways.

Dopamine (DA) is a prominent modulatory neurotransmitter. In all vertebrates, including fish, amphibians, mammals, reptiles, and birds, and it is involved in a broad range of functions, including motor control and motor learning, memory formation, and reward-seeking behavior, among others^1^,^2^,^3^,^4^,^5^. Thus it is not surprising that several properties of dopaminergic systems are shared across vertebrates, including DA biosynthetic and catalytic pathways, many aspects of the organization of DA projection systems, physiological properties of DA neurons, and several aspects of the brain distribution of DA receptors^6^,^7^,^8^,^9^,^10^,^11^,^12^ (see also reviews in^13^). In mammals, synaptic availability of DA is regulated by the kinetics of DA release and, more importantly, through rapid re-uptake by a plasma membrane bound DA transporter (DAT; a.k.a. SLC6A3). Consistent with this prominent physiological role, the DAT gene has been associated with neurological disorders, such as dystonia/parkinsonism^14^, and psychiatric disorders such as attention deficit hyperactivity disorder, bipolar disorder, clinical depression, and drug addiction^15^,^16^. Similar to mammals, there is evidence that synaptic DA re-uptake also operates in birds and reptiles, playing important roles in behaviors such as communication through learned vocalizations in songbirds, control of male sexual behavior in quail^17^,^18^ and visual courtship displays in whiptail lizards^19^,^20^,^21^. The general assumption has thus been that synaptic DA re-uptake is conserved across vertebrates^22^,^23^, however the genetic basis of DA transport systems in non-mammalian amniotes is unknown.

To address this gap, we set out to investigate the evolution and brain expression of the DAT gene in sauropsids (birds and reptiles). We focused on the substantia nigra (SN) and ventral tegmental area (VTA), which are the primary sources of DA projections to the striatum and limbic structures, and are particularly prominent in higher vertebrates like mammals, reptiles and birds (see reviews in^13^). DA cells in both the SN and VTA are readily identifiable through expression of the biosynthetic enzymes dopamine decarboxylase (DDC) and tyrosine hydroxylase (TH). Furthermore, these cells in mammals express DAT, which regulates the synaptic availability of DA and limits its actions on post-synaptic receptors within mesostriatal and mesocorticolimbic DA projections.

Using a combination of sequence alignments and synteny analysis, we discovered that the DAT gene is absent in sauropsids, raising questions concerning how birds and reptiles regulate synaptic DA levels, and whether this basic feature of DA projection systems might differ from mammals. Using *in situ* hybridization, we obtained unambiguous evidence that the noradrenergic transporter (NAT; a.k.a. SLC6A2), which in mammals is a marker of noradrenergic (NA) cells, is expressed in the SN and VTA of birds (Zebra finch) and lizards (Green anole), where it co-localizes with DA cell markers. This pattern is in sharp contrast to mammals, where DAT and NAT segregate to distinct brain nuclei and cell types, and fish, where we found no co-expression of NAT and DAT. We suggest that this modified NAT expression functionally compensates for loss of DAT in birds and reptiles, a possibility that explains previous pharmacological data indicating that avian striatal DA transport has features consistent with NAT of mammals^24^,^25^. Thus, DA transport systems have distinct genetic bases in birds/lizards compared to mammals (usage of NAT as opposed to DAT). These findings yield new insights into the transcriptional regulation of DA cells and, more generally, into the evolution of DA transporter mechanisms in amniotes.

## Materials & Methods

### Phylogenomic Analysis of the DAT Gene

To examine the presence/absence of the DAT gene across the vertebrate phylogeny we first consulted Ensembl (e75) and NCBI’s Refseq collections to identify any entries in the genomes of mammals (i.e. opossum, BOADO5; platypus, OANA5; mouse, GRCm38; human, GRCh37), birds (i.e. chicken, Galgal4; zebra finch, taeGut3.2.4; turkey, UMD2), crocodiles (i.e. American alligator, allMis0.2; Chinese alligator; ASM45574v1), reptiles (Anole lizard, AnoCar2.0; Chinese softshell turtle, PelSin_1.0), amphibians (xenopus; JGI_4.2), and teleosts (i.e. tilapia, Orenil1.0; fugu, FUGU4; zebrafish, Zv9) currently annotated as either SLC6A3 or DAT. We did not find any such entries in any sauropsid (i.e. reptile or avian) genomes. However, to be certain that the DAT gene was not present but rather mis-annotated, or “hiding” in an unassembled region of the genome, we next conducted an exhaustive series of BLAT alignments against well-assembled high-coverage avian genomes (above avian genomes plus medium ground finch, GeoFor_1.0; and budgerigar, melUnd1), crocodile (American alligator, Allmis0.2), and reptile genomes (above reptile genomes plus painted turtle, chrPic1) using both nucleotide and protein coding sequences from DAT orthologs in zebrafish (ENSARG00000004219), frog (ENSXETG00000001728), mouse (ENSMUSG00000021609), and humans (ENSG00000142319). BLAT^26^ searches were conducted using parameters that maximized sensitivity, which increased the number of false positives, but allowed us to verify that the DAT gene was not present. We also performed additional BLAT and BLASTn searches against an improved gap-filled zebra finch genome (Mello and Warren, unpublished data) assembled from additional Illumina sequencing reads, as well as the largest available chicken EST collections (e.g. BBSRC give details here, Univ. Delaware Chick EST). We also conducted a series of BLASTn and BLASTp searches (using Block Substitution Matrix 45 for highly divergent sequences; word size 11 for DNA, 3 for protein) of NCBI’s nucleotide and protein collections. Finally, we conducted comprehensive tBLASTn searches^21^ against unassembled Whole Genome Shotgun (WGS) sequences derived from 45 additional avian genomes (N = 45; Table S1 in^27^; datasets available at http://gigadb.org/dataset/view/id/101000/files_page/2), as well as several others that have been made publically available by various research groups (N = 12; *Puerto Rican parrot, Amazona vittata*; Golden Eagle, *Aquila chrysaetos;* Scarlet macaw, *Ara* macao; Northern bobwhite, *Colinus virginianus;* Hooded crow, *Corvus cornix*; Japanese quail, *Coturnix japonica*; Saker falcon, *Falco cherrug*; Collared flycatcher, *Ficedula albicollis*; Black grouse, *Lyurus tetrix;* Tibetan tit, *Pseudopodoces humilis;* Canary, *Serinus canaria*; White-throated sparrow *Zonotrichia albicollis*). For each of these analyses, we manually verified any significant hits by syntenic analysis whenever possible. We note that in all cases, the highest-scoring hits were to closely related monoamine transporter genes (i.e. SLC6A2, a.k.a. NAT, or SLC6A4, a.k.a. SERT). We note that fish, lizards, and birds possess an additional paralog of SLC6A4 (a.k.a. SLC6A4B) that is absent in mammals, including humans (See Results and for details). We also note that for nearly all of the species examined we were able to directly verify the presence of the chromosomal region that would be expected to contain the DAT gene by conducting a comparative analysis of synteny.

Of note, our search, alignment, and annotation efforts were required to establish the DAT gene loss in sauropsids, and we did not simply rely on the annotations provided by Ensembl. In our experience, Ensembl is an incomplete prediction database. Several genes that are known to exist in a given genome are often incompletely predicted, or not predicted at all. Furthermore, Ensembl contains genes that have been misannotated. As previous studies from our group have revealed^28^,^29^,^30^, cases of gene duplications and/or gene losses can only be conclusively established by utilizing direct searches with appropriate alignment tools such as BLAT and BLAST against the appropriate genomes, and by direct examination of mRNA/EST databases.

All sequence analyses were performed using the eBioX (1.5.1) suite of analysis tools. Multiple alignments of NAT protein sequences were estimated using the ClustalW alignment algorithm with standard parameters^31^, and sequence identities were calculated using SIAS (http://imed.med.ucm.es/Tools/sias.html; gaps were not taken into account).

### Animal preparation

We used a total of 6 adult male zebra finches (*Taeniopygia guttata*), 4 adult sex-undetermined green anoles (*Anolis carolinensis*), and 3 adult male dominant Burtoni cichlids (*Astatotilapia burtoni*) that were respectively obtained from our own breeding colony, purchased from local breeders, or made available to us by the Renn lab (Reed College). For zebra finches, individuals were first isolated overnight (12:12-hour light-dark cycle) in custom-built acoustic isolation chambers to reduce non-specific auditory stimulation. On the following morning (~9:00 AM), birds were monitored for at least 1 hour, and confirmed to be non-singing. Regardless of species, animals were sacrificed by decapitation, their brains were removed and bisected to reproduce an approximation of the “Frankfurt” frontal plane^32^, and frozen in O.C.T. compound (Tissue-Tek). Brains were sectioned on a cryostat (10 μm) and prepared for *in situ* hybridization as described in^33^. Animal protocols were approved by OHSU’s IACUC committee and are in accordance with NIH guidelines.

### Clone Selection and In situ Hybridization

Clones corresponding to the Zebra finch noradrenaline transporter (NAT; FE735918) and dopa decarboxylase (DDC; DV957042) genes were obtained from the ESTIMA Zebra finch brain cDNA collection^34^. For these clones, plasmid DNA was restriction enzyme digested (BSSHII; New England Biolabs) to release the template insert, and then purified with a PureLink PCR purification kit (Life technologies). To obtain Zebra finch dopamine beta-hydroxylase (DBH), we designed primers (Primers: DBHexon1-10, 5′-GCCAGGTGCCGGGTGCCATCC-3′; DBHexon1-313, 5′-GTCCGACATCCCGAAGAGG-3′) to PCR amplify the first exon of the DBH gene from genomic DNA derived from Zebra finch muscle genomic DNA. Gradient PCR (Ta = 42 °C to 62 °C; 30 rounds of amplification) yielded a ~313 bp product, which was then gel purified, and cloned into a pBluescript-II (+) vector (Stratagene). The partial mRNA sequence for this clone has been submitted to GenBank (Accession Number: KP299259).

Clones for the Nile Tilapia (*Oreochromis niloticus*) dopamine transporter (DAT; GR671131) and for NAT (GR666231) were obtained from the Cichlid Genome Consortium (http://cichlid.umd.edu/cichlidlabs/kocherlab/bouillabase.html) and the Kocher lab^35^. Clones for the green anole (*Anolis carolinensis*) NAT (FG784332) and tyrosine hydroxylase (TH; FG660029) were obtained from the Colbourne lab and the Indiana University Center for Genomics and Informatics^36^. Because Nile Tilapia and Green Anole cDNAs were cloned into sequencing vectors that lacked RNA polymerase promoter sites for riboprobe synthesis, we PCR amplified these cDNA using pairs of gene specific primers that included an additional 5′-extension corresponding to the sequence for T3 (antisense strand) or T7 (sense strand) RNA polymerase promoter (Ta = 60 °C, 35 rounds of amplification). Resulting DNA fragments were purified using a PureLink PCR clean-up kit (Life Technologies) and used as template for *in vitro* transcription of cRNA probes (see Supplementary Table 1 for primer and template details). For ESTIMA clones and PCR-derived templates, sense and antisense strand probes were synthesized at 37 °C for 3 hours using the appropriate T3 or T7 RNA polymerase (Promega Inc., Madison, WI), with either digoxigenin (DIG)-UTP or fluorescein-UTP in the nucleotide label mix. Resulting probes were purified by Sephadex G-50 columns^37^.

All of the methods for performing single and double *in situ* hybridization were essentially as described in^30^,^33^,^37^. After post-fixation and dehydration sections were hybridized with a solution (16 μl per section) containing 50% deionized formamide, 2 × SSPE, 1 μg/μl tRNA, 1 μg/μl BSA, 1 μg/μl poly-A in DEPC-treated water, and 2 μl of DIG- and/or fluorescein-labeled riboprobe. Slides were coverslipped, sealed by immersion in mineral oil, and incubated overnight at 63–65 °C. The following day sections were rinsed in chloroform, de-coverslipped in 2x SSPE, and washed by incubating serially for 1 hr at RT in 2 × SSPE, 1 hr at 63–65 °C in 2 × SSPE containing 50% formamide, and twice in 0.1 × SSPE for 30 min at 63–65 °C.

For single-labeling *in situ* hybridization, sections were blocked for 30 min at RT in TNB buffer (100 mM Tris-HCl pH 7.4, 150 mM NaCl, 20 μg/μl bovine serum albumin, 0.3% Triton X-100), incubated for 2 hr in TNB with an alkaline phosphatase conjugated anti-DIG antibody (anti-DIG-AP; 1:600 dil., Roche Applied Science, Mannheim, Germany), washed briefly in TNM buffer (in mM; 100 Tris-HCl pH 9.5, 150 NaCl, 5 MgCl_2_), and incubated for 1–3 days in a ready-to-use tris-buffered solution containing the alkaline phosphatase substrates Nitro-Blue Tetrazolium Chloride (NBT; 0.42 g/L) and 5-Bromo-4-Chloro-3-Indolyl-phosphate p-Toluidine Salt (BCIP/NBT; 0.21 g/L; Substrate Solution NEL937, Perkin-Elmer, Waltham, MA). Developed slides were briefly rinsed in distilled water to remove salts, fixed in 3% buffered paraformaldehyde solution, rinsed in distilled water, and coverslipped with aquamount (Lerner Laboratories, Pittsburg, PA).

For double-labeling fluorescent *in situ* hybridization, we used various combinations of DIG- and fluorescein-labeled riboprobes. All of the hybridizations, washes, and section blocking were essentially as described above for single labeling *in situs*. DIG-labeled probes were developed first by incubating sections overnight at 4 °C in TNB with a horseradish peroxidase conjugated anti-DIG antibody (anti-DIG-HRP; 1:800 dil.; Roche Applied Science). Sections were then washed 3 times for 5 min at RT in TNT (100 mM Tris-HCl pH9.5, 150 mM NaCl, 0.3% Triton X-100), and incubated at RT for 1 hr with Alexa 488-conjugated-tyramide in amplification buffer (1:100 dil.; Invitrogen, Carlsbad, CA) according to the manufacturer’s recommended protocol. Sections were then washed 3 times for 5 min in TNT, and incubated for 20 min at RT in TNT plus 0.2% HCl to inactive the anti-DIG-HRP, followed by three washes for 5 min each in TNT. To visualize the fluorescein-labeled probe, sections were incubated for 2 hr in a solution of TNB with an HRP-conjugated anti-fluorescein antibody (1:500 dil., Roche Applied Science), washed 3 times for 5 min in TNT, and incubated at RT for 1 hr with Alexa 568-conjugated-tyramide in amplification buffer. Finally, sections were washed in TNT, counterstained with propidium iodide (0.1 μg/mL in TNT), and coverslipped with aquamount. For each set of double-labeling *in situs* we included negative controls slides that were incubated without one or the other primary antisera to confirm labeling specificity and to verify HRP inactivation. We also developed in parallel a set of single labeling *in situs* for the DIG- and fluorescein-labeled riboprobes to verify detection sensitivity.

To address the issue of probe specificity, all clones utilized for *in situ* hybridization were examined by BLAT alignments to the corresponding genomes to verify that they specifically aligned to the expected loci, and lacked significant alignments to other loci. Furthermore, controls using sense strand probes or omitting riboprobes altogether were routinely incorporated to the hybridizations, and yielded no detectable signals.

### Image Acquisition and Figure Preparation

Images of non-fluorescent *in situ* hybridization were acquired using a high-resolution digital slide-scanning system (Olympus Nanozoomer HT2). Additional photomicrographs were acquired with bright-field, or fluorescence optics using a digital camera (DVC co., Austin, TX) coupled to a Nikon E600 microscope. Drawings of transverse mouse brain sections were drawn based on Nissl series images available at the Allen Mouse Brain Atlas; available from: http://mouse.brain-map.org/. Photomicrographs showing the expression of NAT, DDC, and DBH in the mouse brain were obtained from the Allen Mouse Brain Atlas [Internet]; available from: http://mouse.brain-map.org (©2014 Allen Institute for Brain Science)^38^. We based our identification of catecholaminergic cell groups on brain atlases for zebra finch^32^,^39^, teleosts^8^,^9^,^40^, and lizard^12^, as well as reviews in^13^. Photoshop-CS5 (Adobe Systems Inc., San Jose, CA) was used to adjust photomicrographs. Specifically, we used the levels function to adjust the contrast and brightness of gray scale and color images. Color balancing was performed across sections so that background levels were similar. Figures were prepared in Illustrator-CS6 (Adobe Systems Inc.). BoxShade (http://www.ch.embnet.org/software/BOX_doc.html) was used to graphically present protein sequence multi-alignments and shade conserved residues.

### NAT and DAT Comparative Promoter Analysis

To determine if the promoters of sauropsid NAT genes might contain transcription regulatory elements that are generally present in mammalian DATs, but not in NATs, we conducted a comprehensive comparative analysis of the NAT (and DAT) promoters in avian (i.e. chicken, zebra finch, turkey, and budgerigar) and mammalian (i.e. platypus, opossum, mouse, and rat) species. We first identified putative general promoter regions by determining a putative transcription start site (TSS) based on the alignments of same species ESTs, mRNAs, and RefSeq gene predictions. We next extracted the genomic sequence for a region ~2.5 kb upstream of the putative TSS; inclusion of sequences further upstream in our analysis was not feasible as these were either highly divergent or missing due to genomic gaps in several species. In some cases we were able to refine, or computationally validate the location of the TSS by scanning each promoter region with algorithms available through Softberry (http://linux1.softberry.om/berry.phtml) that predict TSSs based on the position of TATA or non-TATA promoter sequences (FPROM^41^), or the location of CpG-rich islands (CpGFinder). Next, we used the ‘SCAN’ algorithm available at JASPAR (http://jasper.genereg.net^42^) to identify the positions of putative vertebrate transcription factor binding sites (TFBS^43^). We scanned each promoter sequence with the complete set of vertebrate core TFBS position weight matrix models that are available at JASPAR (http://jaspar.binf.ku.dk/cgi-bin/jaspar_db.pl?rm=browse&db=core&tax_group=vertebrates) using conservative thresholding (i.e. relative profile score threshold >90%). For each lineage (i.e. avian or mammalian), and each gene (i.e. NAT or DAT), we next aligned sets of promoters according to the location of their TSS, and searched for the presence of conserved TFBSs within a series of consecutive ~500 bp segments starting at the TSS. For instance, starting with the first segment of the Zebra finch NAT promoter (bp 0 to −500) we first identified any TFBSs that were also present within the same segment in the budgerigar, chicken, and turkey NAT orthologs; we next sequentially identified the elements present in each of the other four 500 bp segments, up to position −2.5 kb from the TSS. We applied an identical analysis to the set of mammalian NAT and DAT promoters, using mouse NAT and DAT promoters as a starting point for each comparison, which included mouse, rat, opossum, and platypus (not included for DAT due to genomic gaps). All non-conserved TFBSs (i.e. not present in all species examined within each lineage) were removed from the analysis. Finally, we compared the composition of conserved TFBS in the avian NAT promoters (using the position of each TFBS in Zebra finch NAT) with the compositions of the conserved TFBS in the mammalian NAT and DAT promoters, and identified any TFBSs that were shared between the consensus avian NAT with the consensus mammalian NAT or with the consensus mammalian DAT promoters. We noticed that there is a highly conserved ARNT element in the proximal region of all consensus promoters and used this site to align the sequences presented; all other shared elements and the putative TSSs are mapped with reference to that element (i.e. position 0).

## Results

### The dopamine transporter (DAT) gene is missing in sauropsids

**Figure 1:**
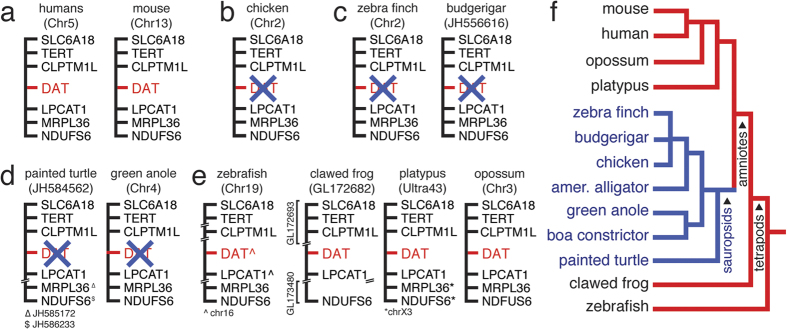
Evidence of the loss of the DAT gene in the vertebrate lineage containing birds and reptiles (i.e. sauropsids). (**a**) Schematic representation of conserved chromosomal loci that contain DAT in representative mammalian species. (**b**–**d**) Lack of the DAT gene in this same conserved region in chicken (**b**), representative neoaves (**c**) and reptilian (**d**) species. (**e**) The DAT gene is found in representative non-sauropsid species, including fish, amphibians and non-eutherian mammals. In a-e, the position of the DAT gene is indicated in red, flanking syntenic genes in black, and missing DAT orthologs by a blue “X”. (**e**) Schematic tree (unscaled) depicting the phylogenetic relationships among representative species of the major tetrapod lineages. Major branches that have retained the DAT gene are indicated in red, those that have lost the DAT gene are indicated in blue.

To determine whether the molecular determinants of DA transport in the vertebrate lineage that contains birds and reptiles (i.e. sauropsids) are as in mammals, we first searched the genomes of birds for the DAT gene. We note that in mammals, the DAT gene is situated within a highly conserved chromosomal region, immediately flanked by CLPTM1L and LPCAT1 (Fig. 1a). To our surprise, we found no evidence of the DAT gene in this location in chicken, (Fig. 1b) the best assembled, highest coverage sauropsid genome. Of note, the corresponding region in chicken (~12 kb), contains no gaps and has been assembled from high quality reads (Supplementary Fig. 1), thus the absence of the DAT gene is not due to low sequence coverage or assembly issues. Absence of the DAT gene in birds was further confirmed by syntenic analysis and exhaustive BLAT and mega-BLASTn searches of other high-coverage well assembled and annotated avian genomes (zebra finch and budgerigar; Fig. 1c), searches of avian expression databases (e.g. mRNA, EST, and RefSeq databases), as well as mega-BLASTn searches of the whole genome shotgun sequences for a large collection of additional avian species representing the majority of branches of the avian phylogenetic tree^27^,^44^; n = 57; see Methods for details).

To determine the phylogenetic origin of the DAT gene loss in vertebrates, we next performed systematic searches and synteny analysis of the DAT gene in the genomes of representative non-avian sauropsids (American alligator, green anole, painted turtle) available in NCBI. However, we found no traces of the DAT gene in these species, neither at the same syntenic position as in mammals (Fig. 1d), nor in any other region of their genomes, We next investigated non-sauropsid vertebrates and verified the presence of DAT in fish, amphibians and non-eutherian mammals, including platypus and opossum (Fig. 1e). These findings point to a DAT loss that most likely occurred in an ancestral sauropsid (Fig. 1f). We note that the closely related gene that encodes the noradrenaline transporter (NAT; a.k.a. SLC6A2) is present in all the vertebrate species examined at its expected syntenic location (Supplementary Fig. 2). Importantly, we found no evidence of paralogous duplications of either the DAT or NAT gene in the genomes of any of the organisms examined. We also note that all genomes examined contain at least one of two copies of the closely related serotonin transporter gene (SERT; a.k.a. SLC6A4A and SLC6A4B in Supplementary Figs 3 and 4). These likely represent an ancestral vertebrate duplication of SERT, since both copies are present in fish, but some lineages have lost one of the copies (e.g., SLC6A4B is present in fish, amphibian, reptiles, birds, and metatherian mammals, but absent in eutherian mammals, including humans; Supplementary Fig. 3; see also^22^,^23^).

### Modified NAT expression compensates for the DAT loss in birds and lizard

**Figure 2:**
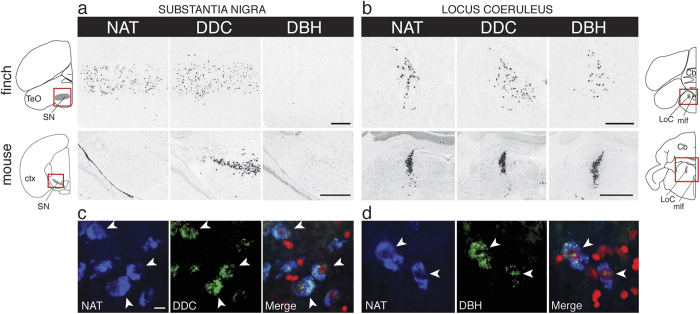
Avian NAT is expressed in both dopaminergic and noradrenergic cell groups. (**a**) *In situ* hybridization reveals that in zebra finch NAT is a marker of the substantia nigra (SN), as shown by the overlapping expression of NAT (top left) with the catecholaminergic marker dopa decarboxylase (DDC; top middle), but not the noradrenergic marker, dopamine beta-hydroxylase (DBH^−^; top-right). In contrast, NAT expression is completely lacking in mouse SN (bottom left), identified here by DDC expression (bottom middle) and lack of DBH expression (bottom right). (**b**) In both zebra finch (top panels) and mouse (bottom panels), NAT is a robust marker of the locus coeruleus, identified here by expression of both DDC and DBH. The locations of photomicrographs are indicated by red rectangles within the drawings of transverse sections through the midbrain (**a**, far left) and pons (**b**, far right) of zebra finch (top) and mouse (bottom). Drawings of transverse mouse brain sections were drawn by hand based on Nissl series images available at the Allen Mouse Brain Atlas; available from: http://mouse.brain-map.org/. Photomicrographs showing the expression of NAT, DDC, and DBH in the mouse brain were obtained from the Allen Mouse Brain Atlas [Internet]; available from: http://mouse.brain-map.org (©2014 Allen Institute for Brain Science) (**c**,**d**) Fluorescent double *in situ* hybridization in zebra finches reveals that NAT expression co-localizes with DDC in the substantia nigra (**c**) and with DBH in the locus coeruleus (**d**), demonstrating its expression in both DA and NA cells. Anatomical abbreviations: ctx, cortex; Cb, cerebellum, LoC, locus coeruleus, mlf, medial longitudinal fasciculus; SN, substantia nigra; TeO, optic tectum. Scale bars = 500 μm in (**a,b**); 10 μm in (**c,d**).

It is possible that the DAT gene loss might be compensated by the modified expression of another monoamine transporter. To test this hypothesis, we used locus specific molecular probes and *in situ* hybridization to map the brain distributions of NAT and SERT along with markers of serotonergic (i.e. tryptophan hydroxylase, TPH2), catecholaminergic (i.e. dopa decarboxylase, DDC, or tyrosine hydroxylase, TH), and noradrenergic (i.e. dopamine β-hydroxylase, DBH) cell groups in a representative avian species (i.e. zebra finch). As in mammals, SERT was expressed in serotonergic nuclei of the raphe complex, but not in the major midbrain dopaminergic nuclei VTA and SN (not shown). Also as expected, NAT was expressed in noradrenergic (NA) nuclei, overlapping with the distribution of DBH (LoC shown in Fig. 2b, top row). Strikingly, however, NAT was also strongly expressed in the dopaminergic nuclei of the midbrain tegmentum, as demonstrated by its overlapping distribution with the catecholaminergic marker DDC (for SN, see Fig. 2a, top row, left and middle panels; for VTA, see Supplementary Fig. 5, top row, left and middle panels). Importantly, these dopaminergic nuclei do not contain NA cells, as confirmed here based on the lack of expression of DBH (Fig. 2a, top row right, and Supplementary Fig. 3, top row right). This finch NAT pattern is in sharp contrast to mammals, where NAT is expressed in noradrenergic nuclei like the LoC (Fig. 2b, bottom row), but not in dopaminergic nuclei, including the SN (Fig. 2a, bottom row) and VTA (Supplementary Fig. 5, bottom row). Furthermore, fluorescent double *in situ* hybridization revealed that NAT expression in finch co-localizes at the cellular level with DDC in both the SN (Fig. 2c) and VTA (not shown), demonstrating its expression in DA cells in these nuclei, and with DBH in the LoC (Fig. 2d), confirming its expression in NA cells in the latter. Thus, the NAT gene in zebra finch is clearly expressed in both DA and NA nuclei and cells, contrary to what is seen in mammals. In all other respects, however, the NAT expression seen in finches was consistent with that seen in mammals (i.e. expression restricted to brainstem cell groups, but completely absent in telencephalic and diencephalic regions; see Supplementary Fig. 6, and compare with image 73615562_204 and 73615562_108 of the Allen’s Institute Mouse Brain atlas (http://mouse.brain-map.org), further attesting to probe specificity and broad conservation of the NAT expression pattern.

**Figure 3:**
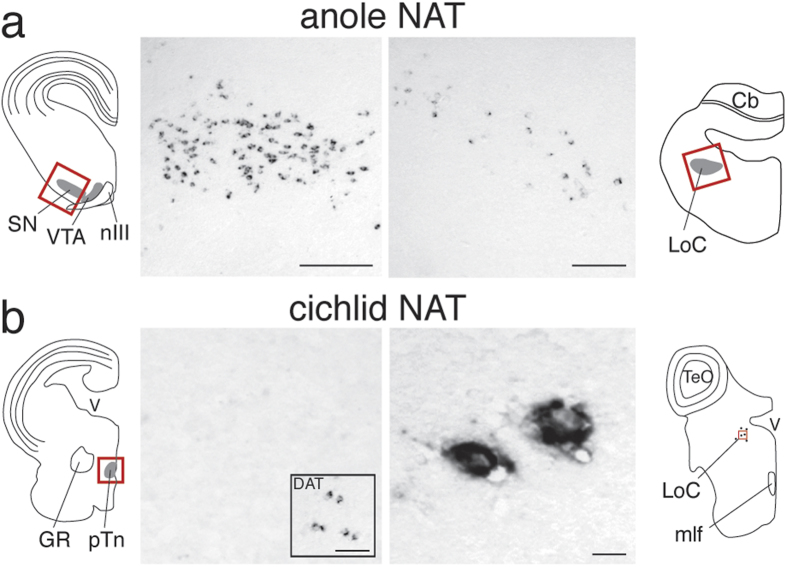
Segregation of NAT and DAT with dopaminergic and noradrenergic cell groups in teleosts, but not reptiles. (**a**) *In situ* hybridization reveals that in green anoles NAT is expressed in the substantia nigra (SN; left) and in the locus coeruleus (LoC; right), consistent with the pattern in finches. (**b**) In cichlid fish, NAT is expressed in the LoC (**b**, right), but not in the posterior tuberculum (pTn; left), which contains the teleost equivalent of striatal-projecting dopaminergic cells in VTA/SN. Presence of dopaminergic cells in the pTn is confirmed here by the inset photomicrograph, showing that cells expressing the teleost DAT ortholog are present in the pTn. The locations of photomicrographs are indicated by red rectangles within the camera lucida drawing of transverse brain sections of the green anole (**a**) and Burtoni cichlid (**b**) at the level of the midbrain tegmentum (left), and locus coeruleus (right). Anatomical abbreviations: Cb, cerebellum; GR, corpus glomerulosum pars rotunda; LoC, locus coeruleus; mlf, medial longitudinal fascicle; nIII, oculomotor nerve; pTn, posterior tuberculum; SN, substantia nigra; VTA, ventral tegmental area. Scale bars = 100 μm in a; 50 μm in b; 500 μm in b, DAT inset.

To determine whether the pattern of NAT expression is unique to birds or extend to other sauropsids, we next analyzed NAT gene expression in the brain of a lizard (i.e. green anole). As in finches, we found that NAT-expressing cells occur in both dopaminergic (SN/VTA) and noradrenergic (LoC) nuclei (Fig. 3a). Importantly, the SN and VTA in lizard are known to lack noradrenergic cells, based on the absence of immunolabeled somata for noradrenergic markers^11^. Combined, our results indicate that the expression of NAT in both DA and NA cells is a general trait of sauropsids.

### Expression of NAT and DAT are segregated in fish

Since fish represent a more basal vertebrate lineage that has both NAT and DAT, we next examined the brain of a representative teleost (i.e. Burtoni cichlid) to determine if the distribution of NAT in fish is comparable to that observed in sauropsids or in mammals. We found that the nucleus of the posterior tuberculum, the teleost equivalent of striatal-projecting cells in VTA/SN^13^, was entirely devoid of NAT expression (Fig. 3b; left panel), although as expected neurons within this nucleus were well labeled by DAT (Fig. 3b, inset), consistent with their DA identity. Also as expected, NAT was strongly expressed in cells within the LoC (Fig. 3b; right panel), consistent with their NA identity, however no DAT expression was found in this nucleus. This pattern in teleosts is consistent with that seen in mammals, where the expression of NAT and DAT is segregated and NAT is not expressed in the SN/VTA, as well as previous evidence of lack of NAT expression in DA cells in medaka^45^. Together, these results indicate that the segregation of DAT with DA cells, and NAT with NA cells is a trait conserved in fish and mammals that was altered in sauropsids, in concert with the loss of the DAT gene.

### Comparative analysis of the NAT and DAT promoters

**Figure 4:**
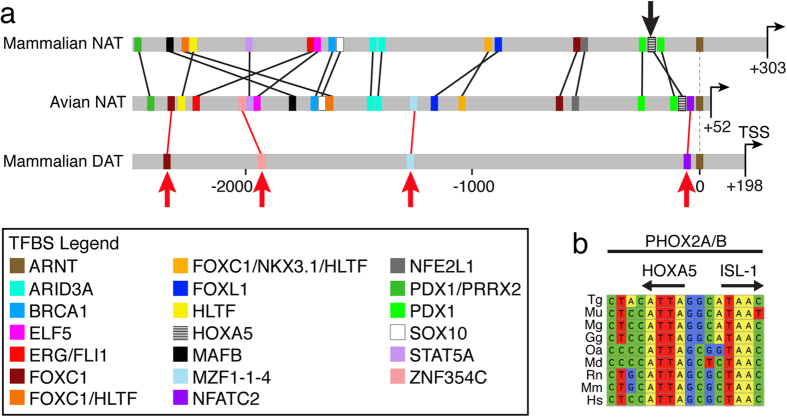
Comparative analysis of NAT and DAT *cis*-regulatory promoter elements. (**a**) Transcription factor binding sites (TFBSs; colored vertical rectangles) that are conserved in avian NAT (middle line) compared with mammalian NAT (top line) or DAT (bottom line). Shown are ~2,500 kb upstream of the putative transcription start sites (TSSs, thin horizontal arrows), and only the TFBSs that are conserved in all organisms examined within each lineage and that are shared between avian NAT and mammalian NAT (black connecting lines), or avian NAT and mammalian DAT (red connecting lines and red arrows; see Methods for details). Promoters are aligned with respect to the position of the zebra finch and mouse ARNT (indicated by thin dashed line), a conserved site immediately upstream of the putative TSSs in all NAT and DAT promoters. The black vertical arrow indicates the position of a homeodomain-binding motif known to confer cell-type specific expression to human NAT in noradrenergic neurons^59^ and found to be conserved and shared between avian and mammalian NAT, and not mammalian DAT promoters. Color key to all TFBSs is given by legend on bottom left. (**b**) Genomic alignments of a short stretch of the NAT promoter derived from a set of representative species indicates that the core human NAT proximal homeodomain motif that contains binding sites for HOXA5, ISL1 and PHOX2A/B, is conserved across vertebrate phylogeny. Species name abbreviations: Tg, *Taeniopygia guttata*, Mu; *Melopsittacus undulatus*, Mg, *Meleagris gallopavo;* Gg, *Gallus gallus*; Oa; *Ornithorhynchus anatinus*; Md, *Monodelphis domestica*; Rn, Rattus norvegicus; Mm, Mus musculus; Hs, *Homo sapiens*.

In an effort to identify regulatory elements that might be involved in the distinct expression of avian NAT in dopaminergic cells, we performed a comparative analysis of cis-regulatory elements in the promoters of avian NAT, and mammalian NAT and DAT. We specifically searched for conserved transcription factor binding sites based on JASPAR position-weight matrices located within 2,500 bp immediately upstream of the transcription start site of the NAT gene in avian species, and then assessed whether the same conserved elements were also conserved in the promoters of mammalian NAT and/or DAT (see Methods for details). Our primary hypothesis was that despite an overall greater similarity between avian and mammalian NAT genes, the avian NAT would share some specific elements with mammalian DAT that are absent in mammalian NAT. Such elements, if present, might contribute to NAT expression in DA cells and thus constitute important candidates for mechanistic studies of gene expression regulation in DA cells. As expected, we found that the avian NAT promoter region shares a large number of conserved elements with mammalian NAT (Fig. 4, shared elements indicated by black lines) that were predictably absent in the mammalian DAT, including the presence of a core proximal homeodomain-binding motif that is known to confer cell type-specific expression of human NAT through interactions with the transcription factors HOXA5 and PHOX2A^46^ (Fig. 4, indicated by black arrow). However, we also found that avian NAT and mammalian DAT, but not mammalian NAT, share the presence of binding sites within similar regions for NFATC2, MZF1 and ZNF354C, as well as for FOXC1 (Fig. 4; red arrows and red lines). Thus, some specific elements that are conserved in mammalian DAT promoters are also present and conserved in avian NAT, representing candidate elements that may relate to expression in DA cells.

## Discussion

We present conclusive evidence that the DAT gene is missing in sauropsids. Thus, a central component of the dopaminergic system that has traditionally been viewed as being highly conserved among vertebrates is actually absent in a major vertebrate lineage. Furthermore, we have established that in organisms that lack DAT, the NAT gene is expressed in DA cells. This expression pattern is in stark contrast with that observed in mammals, and provides a likely mechanism of compensation for the DAT gene loss. Importantly, we provide evidence that it is the sauropsid NAT ortholog that is expressed in DA cells, not a close paralog of the DAT or NAT genes, for which there is presently no evidence in sauropsids or in any other vertebrate genomes analyzed to date.

**Figure 5:**
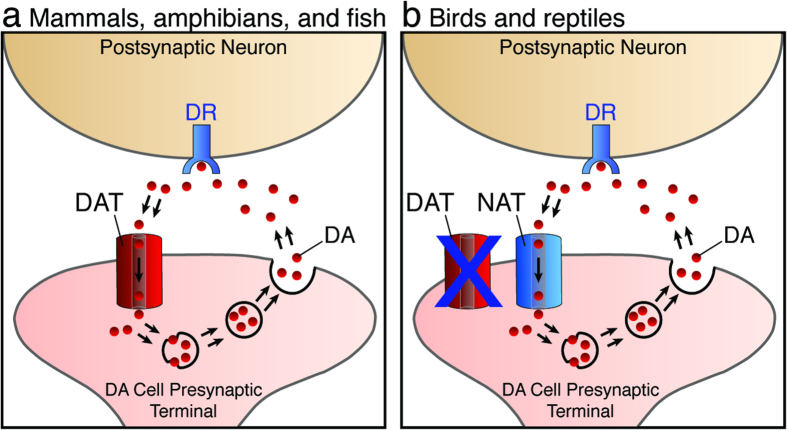
NAT expression in dopaminergic cells of birds and reptiles compensates for the loss of DAT. (**a**) In mammals, amphibians, and fish, the dopamine transporter (DAT) regulates the synaptic availability of dopamine (DA, red circles) through direct reuptake, thus limiting the action of DA on post-synaptically expressed dopamine receptors (DR, corresponding to either D1 and D2, depending on the specific cell type and pathway considered). (**b**) In birds and reptiles, the noradrenaline transporter (NAT), which is functionally capable of DA reuptake, is expressed in DA cells, providing compensation for the loss of the DAT gene.

The loss of the DAT gene was a major surprise, since most features of the DA system are largely conserved across vertebrate taxa. DA transport activity, in particular, plays a key role in limiting synaptic DA availability (Fig. 5a), thus exerting strong modulatory roles over a wide range of functions, including motor control and reward mechanisms. As such, it has been largely assumed that the DAT gene must also be present in organisms like birds and lizards. Indeed, previous experimental evidence detected the presence of DA re-uptake activity in the mesostriatal system of the zebra finch^24^,^25^, in terminals from dopaminergic projections from the SN and VTA to striatal song nucleus area X^47^, suggesting that the expression of the DAT gene in SN/VTA was also conserved in sauropsids. In retrospect, however, some important pharmacological properties of the DA re-uptake activity detected in finch striatum notably differ from those in mammals. For instance, it was shown to be sensitive to a DA blocker but at a very high concentration, under which condition the blocker is known to also inhibit NAT, and it shows sensitivity to selective NAT blockers^24^,^25^. Furthermore, the addition of the DAT blocker did not affect the activity beyond the blockade achieved with the NAT inhibitor alone. Given our current findings, we conclude that the DAT activity detected in these previous studies in finches was due to the expression of NAT in DA cells (Fig. 5b). Thus, our data provide a genetic basis for a characteristic feature of the DA re-uptake activity seen in a species that lacks a DAT gene. Of note, mammalian NAT has previously been shown to be capable of DA re-uptake^48^,^49^,^50^,^51^, in fact even more so than DAT itself, and is thought to be responsible for synaptic DA re-uptake in mammalian cortical regions that receive noradrenergic innervation^52^. Thus there is precedence for NAT playing a role in DA regulation, but this has never been reported as occurring through the expression of NAT in DA cells. Importantly, the avian NAT sequences are largely conserved with those in fish and mammals (Supplementary Fig. 7), and share higher amino acid identities with all orthologs of NAT compared to those of DAT (Supplementary Fig. 8). Moreover, the region of the chicken NAT protein that is predicted to encode all 12 transmembrane domains and extracellular loops (Supplemental Fig. 7) shares 98.8%, 90.7%, and 90.5.1% amino acid identity and 99.2%, 94.5%, and 94.5% amino acid similarity with orthologs in zebra finch, mouse, and human. In contrast, chicken NAT shares only 71.6% identity, and 83.1% similarity with human DAT. Furthermore, nearly every amino acid residue that has been linked to DA re-uptake activity based on analyses of site-directed mutations and chimeras^53^,^54^,^55^ is not only conserved in mammalian DAT orthologs, but also in both mammalian and avian NAT orthologs. Thus, like mammalian NAT, sauropsid NAT would be predicted to be capable of the re-uptake of both NA and DA.

Our study also provides insights into the phylogenetic origin of the DAT gene loss and of its apparent compensation via NAT. Both the NAT and DAT genes are clearly present in mammals and in outgroups to the amniotes, including amphibians and fish. They derive from an ancestral vertebrate duplication of an invertebrate monoamine transporter gene (MAT) with broad substrate specificity^22^,^23^, which likely explains their shared ability to transport NA and DA. A previous study reporting a failure to clone a DAT brain transcript in a teleost, the medaka, had suggested that the gene might be missing in that species^45^, but the study was conducted before the medaka genome was available. It is now clear that the DAT gene is present in medaka as well. In contrast, we found that the DAT gene, which is present in mammals, is absent in all sauropsids analyzed (including lizard, turtle, snake, crocodile, and birds). The most parsimonious explanation for this absence is the loss of the DAT gene in an ancestral sauropsid. While it is unclear how this loss occurred, it does not appear to have resulted from a major chromosomal rearrangement, since the local syntenic regions that flank the DAT gene are conserved across phylogeny, from fish to humans and, except for the missing DAT gene, also in sauropsids. Also interestingly, this region does not seem to be particularly enriched in repetitive elements. For instance, we find only one Long interspersed nuclear element (LINE) and one Long terminal repeat element (LTR) within the 15 kb region that should contain the DAT gene (not shown). Importantly, the expression of NAT in DA cells is unique to sauropsids, since the expression of NAT and DAT in both mammals and fish is segregated into NA and DA cells, respectively (our present data, which are consistent with a lack of NAT expression in DA cells in medaka^45^). These findings indicate that the segregated expression of these transporters is a basal condition in vertebrates, whereas the expression of NAT in DA cells is a uniquely sauropsid trait.

Our findings raise several questions about gene expression regulation in DA cells, most intriguingly how the modified expression of the NAT gene is accomplished in sauropsids. One possibility is that the regulatory region of NAT differs between birds/reptiles vs. mammals, accounting for the divergent expression modes. Under this scenario, the NAT promoter in sauropsids would contain *cis*-regulatory elements characteristic of mammalian DAT, which would allow for the regulated expression of NAT in DA cells. We found that the avian NAT promoter region resembles closely the mammalian NAT promoter, including the shared presence of a core proximal homeodomain-binding motif known to confer cell type-specific transcriptional regulation of human NAT through interactions with the transcription factors HOXA5 and PHOX2A^46^. However, we also found that avian NAT shares with mammalian DAT a few motifs that are absent in mammalian NAT. Among these, MZF1 and NFATC have previously been linked to regulation of phenotypic features of dopaminergic cells^56^,^57^. Thus, while the evidence does not broadly support a close similarity between the sauropsid NAT and mammalian DAT promoters, it points to some interesting candidate elements worth of further study. We also note that other DAT-related regulatory elements such as enhancers^58^,^59^ could be located upstream of the NAT promoter in sauropsids, and thus confer NAT expression in DA cells of this lineage. Alternatively, the key to cell-specific regulation might lie in DA cells in sauropsids (and not in mammals) expressing TFs (e.g. PHOX2A) that allow for NAT expression in DA cells^59^. Further analyses aimed at distinguishing among these possibilities should considerably deepen our understanding of factors that are critical for regulating gene expression in DA cells of vertebrates.

Our current findings also raise questions about whether other molecular and functional features of dopaminergic systems are conserved or not when comparing mammals vs. reptiles/birds. This is an important issue, given that DA and associated mechanisms are thought to play broad roles in basic functions such as the learning and execution of complex motor sequences and the expression of reward-seeking behaviors. Given the evidence for compensatory NAT expression in DA cells, it appears that DA uptake function is largely conserved in sauropsid and mammals. Of note, both DAT and NAT are major targets of cocaine and methamphetamine^15^,^60^,^61^. Since the NAT sequence in birds and reptiles is conserved with mammals, one would predict that despite the absence of the DAT gene, sauropsids would also be susceptible to these drugs if exposed to them. To our knowledge this possibility has not been tested, even though birds are useful experimental models for other drugs of abuse (e.g.^62^,^63^,^64^,^65^). Lastly, the mechanisms underlying vocal learning and production have been widely studied in avian species, particularly songbirds. The extent to which the modulatory actions of dopaminergic circuits onto vocal acquisition and production pathways might be affected by the differences we have uncovered remains to be determined in future studies.

## Additional Information

**How to cite this article**: Lovell, P. V. *et al.* Living without DAT: Loss and compensation of the dopamine transporter gene in sauropsids (birds and reptiles). *Sci. Rep.* **5**, 14093; doi: 10.1038/srep14093 (2015).

## Supplementary Material

Supplementary Information
