# Supplementary material for: Living without DAT: Loss and compensation of the dopamine transporter gene in sauropsids (birds and reptiles)
Source: Sci Rep. 2015 Sep 14;5:14093. doi: 10.1038/srep14093 (PMC4894405; doi:10.1038/srep14093)
Supplement: Supplementary Information [file srep14093-s1.pdf]

Supplementary Information

Living without DAT: Loss and compensation of the dopamine transporter gene in sauropsids (birds and reptiles).

Lovell, P.V.[1], Kasimi, B.[1,2], Carleton, J.[1,3], Velho, T.A.[4], and *Mello C.V.[1]

[1] Department of Behavioral Neuroscience; Oregon Health & Science University; Portland, OR 97239-3098; USA
[2] Department of Biology; Portland State University; Portland, OR 97207-0751; USA
[3] Molecular Biology Program; University of Utah; Salt Lake City, UT 84112-9057; USA
[4] Department of Neurobiology; University of Massachusetts Medical School, Worcester, Massachusetts 01655; USA

* Corresponding Author
Claudio V. Mello
Department of Behavioral Neuroscience
Oregon Health & Science University
Portland, OR 97239-3098
(503)-418-2650

Supplementary Table 1. Primer pairs used for the amplification of TH, NAT, and DAT from cloned cDNAs derived from Nile Tilapia (*Orechromis niloticus*), and Green Anole (*Anolis carolinensis*). T3 or T7 RNA polymerase promoter sequences were added to gene specific primer pairs as linkers and the resulting PCR products were gel purified and used as DNA templates for DIG-labeled riboprobe synthesis (see Methods for details).

Supplementary Figure 1. Comparative analysis of the DAT locus in the human and chicken genome assemblies. (a) The highly conserved region on human chromosome 5 (Assembly GRCh37/hg19; UCSC browser) that contains the DAT gene (region indicated by vertical dotted lines) is flanked by CLPTM1L and LPCAT1. Ensembl transcript variants for these genes, and large intergenic non-coding RNA (RP11-325I22.2) have been removed for clarity. (b and c) The corresponding region on chromosome 2 in chicken (galgal4 2011 assembly in b, galgal3 2006 assembly in c) is flanked by CLPTM1L and LPCAT1, is devoid of genomic gaps (black bars in Gap Locations tracks in a-c), is

comprised of high quality nucleotide sequencing reads (see sequencing quality track in c), and lacks the DAT gene.

Supplementary Figure 2. Schematic representation of conserved chromosomal loci that contain the NAT gene in representative mammalian (a), avian (b), reptilian (c), amphibian (d), and teleost species (e). The position of the NAT gene is indicated in red, and flanking syntenic genes in black.

Supplementary Figure 3. Schematic representations of conserved chromosomal loci for SERT paralogs in representative vertebrate species. (a) SLC6A4A (named SLC6A4 in mammals) and (b) SLC6A4B. Note that SLC6A4B is present in metatherian, but not eutherian mammals, including humans. The position of the SERT paralogs are indicated in red, flanking syntenic genes are indicated in black; missing SERT copies are indicated by a blue “X”.

Supplementary Figure 4. Alignment of the predicted amino acid sequence of SERT paralogs (SLC6A4A and SLC5A4B) from representative vertebrate species. Species name abbreviations are as follows: Dr, *Danio rerio*; Ol, *Oryzia latipes*; Gg, *Gallus gallus*; Hs, *Homo sapiens*. The numbers on the left indicate the relative position of amino acid residues in the respective sequences. NCBI protein and Ensembl Gene accession IDs for the aligned sequences shown are as follows: **SLC6A4A**: Dr, NP_001035061; Ol, ENSORLG00000013932; Gg, ENSGALG00000004246; Hs, ENSG00000108576; **SLC6A4B**: Dr, NP_001170930; Ol, ENSORLG00000007234; Gg, ENSGALG00000005334. Note that model ENSGALG00000005334 was extended by aligning NP_001170930 to the appropriate locus and translating the corresponding chicken genomic sequence.

Supplementary Figure 5. The Avian NAT gene is expressed in the ventral tegmental area (VTA). *In situ* hybridization reveals that in zebra finch NAT is a marker of the VTA, as shown by the overlapping expression of NAT (top left) with the catecholaminergic marker dopa decarboxylase (DDC; top middle), but not with the noradrenergic marker, dopamine beta-hydroxylase (DBH; top-right). In contrast, NAT expression is completely lacking in mouse VTA (bottom left), identified here by DDC expression (bottom middle) and lack of DBH expression (bottom right). The locations of photomicrographs are indicated by red rectangles within the drawings of transverse sections through the midbrain of zebra finch (top, far left) and mouse (bottom, far left). Drawings of transverse mouse brain sections were drawn based on Nissl series images available at the Allen Mouse Brain Atlas; available from: http://mouse.brain-map.org/. Photomicrographs showing the expression of NAT, DDC, and DBH in the mouse brain were obtained from the Allen Mouse Brain Atlas [Internet]; available from: http://mouse.brain-map.org (©2014 Allen Institute for Brain Science) Anatomical abbreviations: ctx, cortex; NIII, oculomotor nerve; SNc, substantia nigra pars compacta; TeO, optic tectum; thal, thalamus; VTA, ventral tegmental area. Scale bars = 500 μm.

Supplementary Figure 6. The *in situ* probes used to detect the avian NAT and DBH genes in zebra finches are highly specific. NAT expression is restricted to midbrain and brainstem cell groups, including Substantia Nigra (SN; top left panel) and locus coeruleus (LoC; bottom left panel), but is completely absent in telencephalic and diencephalic regions. DBH is selectively expressed in the noradrenergic neurons in LoC (bottom right panel), but is not expressed in dopaminergic neurons of the SN, or in any telencephalic/diencephalic regions.

Supplementary Figure 7. Alignment of the predicted amino acid sequence of the chicken NAT gene with orthologs from other species. The numbers on the left indicate the relative position of amino acid residues in the respective sequences. For the corresponding region predicted to contain all 12 transmembrane domains and extracellular loops (i.e. human DAT amino acids 70 – 600) chicken NAT

shares 98.8%, 90.7%, and 90.5.1% amino acid identity with orthologs in zebra finch, mouse, and human. Putative membrane spanning regions (indicated with gray bars) were obtained from the PDB_TM database[1,2].

Supplementary Figure 8. Pairwise comparisons of amino acid identities for NAT and DAT orthologs ortholog protein sequences derived from representative teleosts, reptiles and birds (sauropsids), and mammals. Protein sequences obtained from NCBI and Ensembl were aligned via Kalign multiple alignments (in eBioX), and percent amino acid identities were calculated without gap penalties in SAIS (http://imed.med.ucm.es/Tools/sias.html). Note that NAT protein sequences found in sauropsids share higher amino acid identities with other orthologs of NAT, than with DAT orthologs.

References

1 Kozma, D., Simon, I. & Tusnady, G. E. PDBTM: Protein Data Bank of transmembrane proteins after 8 years. *Nucleic Acids Res* **41**, D524-529, doi:10.1093/nar/gks1169 (2013).

2 Tusnady, G. E., Dosztanyi, Z. & Simon, I. PDB_TM: selection and membrane localization of transmembrane proteins in the protein data bank. *Nucleic Acids Res* **33**, D275-278, doi:10.1093/nar/gki002 (2005).

Supplementary Table 1. Primer pairs used for the amplification of TH, NAT, and DAT from cloned cDNA's derived from Nile Tilapia (*Orechromis niloticus*), and Green Anole

Organism	Gene	GenBank ID	Forward (5'-3')	Reverse (5'-3')
Green Anole				
	TH	FG660029	GCGTAATACGACTCACTATAGGGCG AAAACATTGGGACAACTTGAGGA	GCGCAATTAACCCTCACTAAAGGGAA CTGGCAGCAATAGCAGCAATA
	NAT	FG784332	GCGTAATACGACTCACTATAGGGCG AACGTCGTCTTCGATGATCCTT	GCGCAATTAACCCTCACTAAAGGGAA CTGAACCACTGCAGGAAGATG
Nile Tilapia				
	NAT	GR666231	GCGTAATACGACTCACTATAGGGCG AATGATTCAAGAGTATGCCTGCTAA C	GCGCAATTAACCCTCACTAAAGGGAA CTCACACAACACAAATACAAAGCTG
	DAT	GR671131	GCGTAATACGACTCACTATAGGGCG AACGACCAATGAAAGCTCCAAG	GCGCAATTAACCCTCACTAAAGGGAA CAACTGGCGTTGAGCTCATGT

Supplementary Figure 1

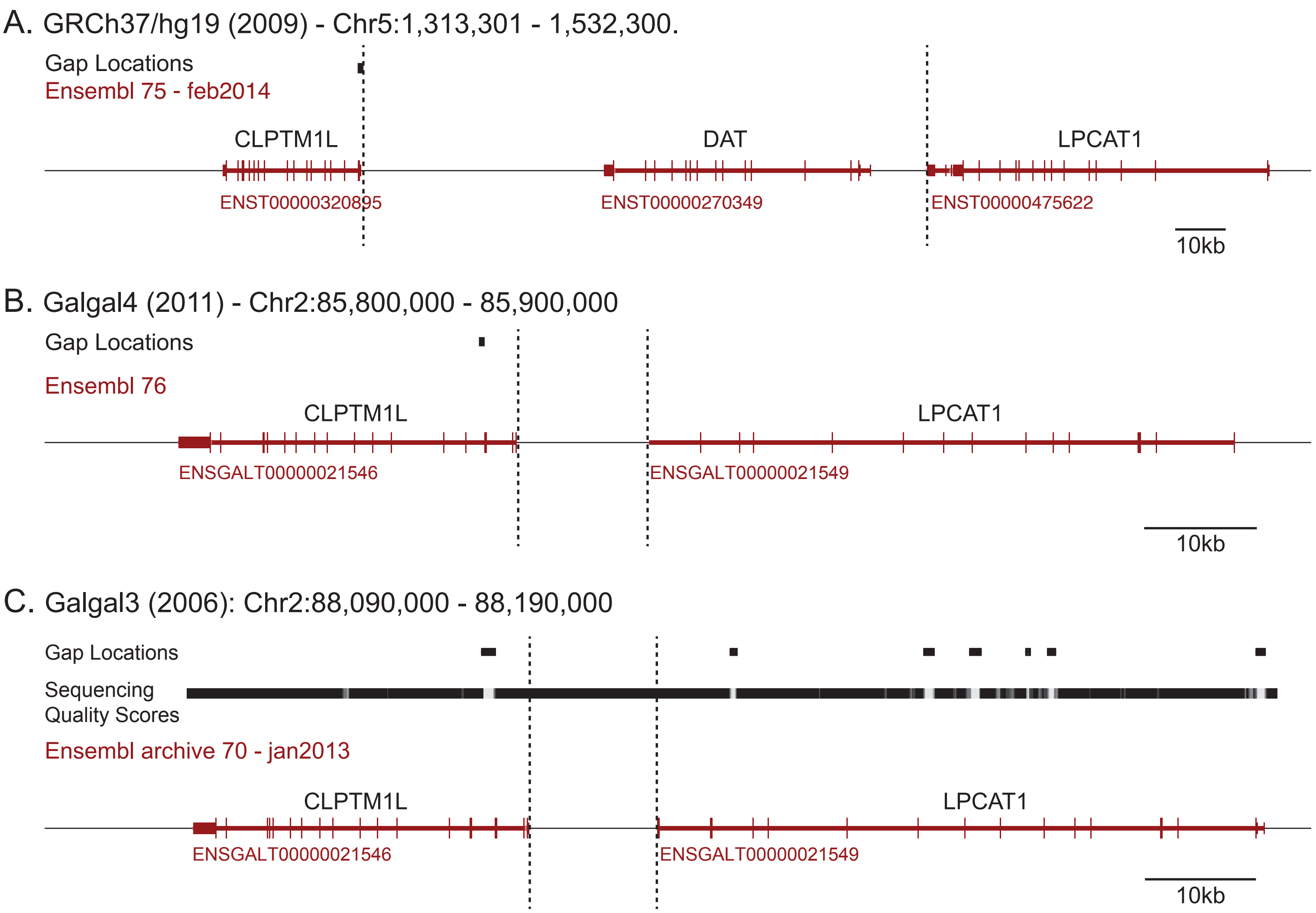

Supplementary Figure 2

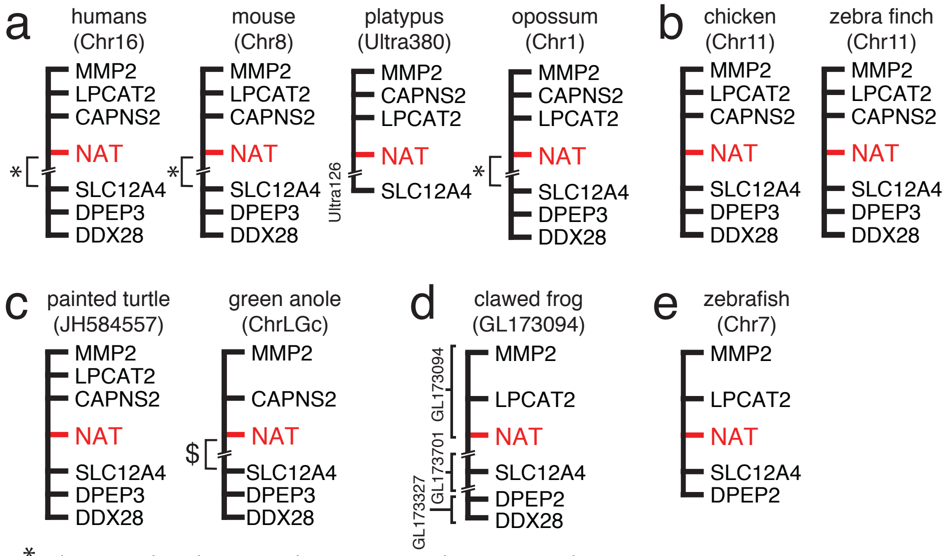

* - denotes an intrachromosomal rearrangment unique to mammals.

$ - denotes an intrachromosomal rearrangment in lizards; not present in turtle.

Supplementary Figure 3

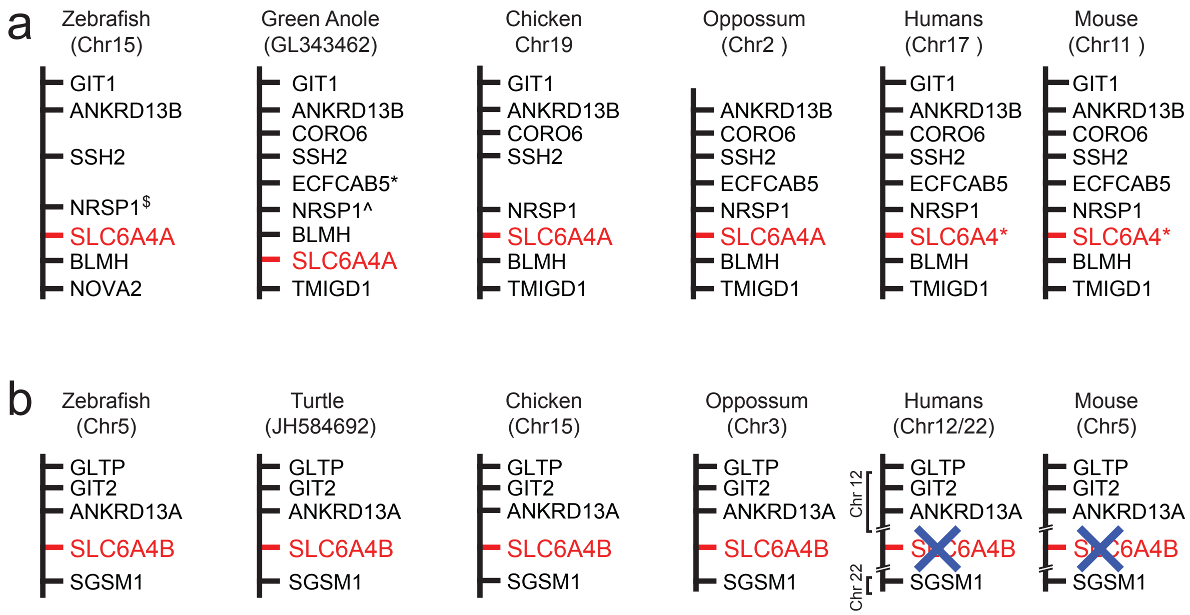

$ Placement inferred from the Nile tilapia (oreNil2)
^ Placement inferred from Painted Turtle (v3.0.1/chrPic1)
* Ortholog of SLC6A4A found in other vertebrates

Supplementary Figure 4

```
Dr_SLC6A4A    1 ----------------------------MDMKESMMMNQEYGGEQQKVPESQENGRLVVD
Ol_SLC6A4A    1 ------------------------------------------------------------
GG_SLC6A4A    1 MENKATSNETQPLTSKKGISDCNEGEDCKENGLLIRNPKSALRLVDDGNKVHPGQGDKEE
Hs_SLC6A4A    1 ----------------------------------------METTPLNSQKQLSACEDGED
Dr_SLC6A4B    1 ------------------------------------------------------------
Ol_SLC6A4B    1 ------------------------------------------------------------
Gg_SLC6A4B    1 ------------------------------------------------------------

Dr_SLC6A4A   33 SVPEKDQKSGSGPGQVSNGYRSTSPQSPKEGAGTGTDRVNTPGTFRTLVVQQTSLDPPRE
Ol_SLC6A4A    1 ------FTTSTPQSPREALGTAVGAVGMPPASGGDTGASAPFGGLRTLVVQQTSLDRPRE
GG_SLC6A4A   61 AAQISNGYSGVQSSVPCSGMAEAEDAQCTAPAATTTTTTTTSTTCGAEGQQQLMELGDRE
Hs_SLC6A4A   21 CQENGVLQKVVPTPGDKVESGQISNGYSAVPSPGAGDDTRHSIPATTTTLVAELHQGERE
Dr_SLC6A4B    1 ---------------------MPHQEQVIAHGNLCAPGPPNNAGYNSNPVPVIIQTESRD
Ol_SLC6A4B    1 ---------------------------MAGSLAHQGSPNPGYNAHNPVTVPVPTQTDSRD
Gg_SLC6A4B    1 ------------------------------------------------------------

Dr_SLC6A4A   93 TWSKKMDFLLSVIGYAVDLGNVWRFPYICYQNGGGAFLLPYLLMAVFGGVPLFYMELALG
Ol_SLC6A4A   55 TWSKKMDFLLSVIGYAVDLGNVWRFPYICYQNGGGAFLLPYLLMAVFGGVPLFYMELALG
GG_SLC6A4A  121 TWSKKIDFLLSVIGYAVDLGNVWRFPYICYQNGGGAFLIPYTIMAIFGGIPLFYMELALG
Hs_SLC6A4A   81 TWGKKVDFLLSVIGYAVDLGNVWRFPYICYQNGGGAFLLPYTIMAIFGGIPLFYMELALG
Dr_SLC6A4B   40 KWSKKMDFLLSVIGFAVDLGNVWRFPYICYQNGGGAFLIPYVLMAVFGGVPLFYMELALG
Ol_SLC6A4B   34 KWSKKMDFLLSVIGFAVDLGNVWRFPYICYQNGGGAFLIPYILMAIFGGVPLFYMELALG
Gg_SLC6A4B    1 KWSKKMDFLLSVVGFAVDLGNVWRFPYICYQNGGGAFLIPYTLMAVFGGVPLFYMELALG

Dr_SLC6A4A  153 QFHRSGCISIWKHVCPIFKGIGFAICIIALYIAFYYNTIMAWALYYLLSSFRATLPWTTC
Ol_SLC6A4A  115 QFHRSGCISIWKHICPIFKGIGFAICIIALYIAFYYNTIMAWALYYLLSSFRPTLPWTTC
GG_SLC6A4A  181 QYHRNGCISIWRKICPIFKGIGFAICIIDLYVASYYNTIMAWVFYYLVSSFTTELPWTSC
Hs_SLC6A4A  141 QYHRNGCISIWRKICPIFKGIGYAICIIAFYIASYYNTIMAWALYYLISSFTDQLPWTSC
Dr_SLC6A4B  100 QFHRTGAISIWKHICPIFKGIGFAICIIALYVSFYYNTIIAWALFYFYSSFSSTLPWTSC
Ol_SLC6A4B   94 QFHRTGAISIWKHICPIFKGIGYAICIIALYVSFYYNTIIAWALFYFYSSFSTILPWTNC
Gg_SLC6A4B   61 QFHRTGAIPIWKHICPIFKGIGFAICIIGLYVSFYYNTIIAWALYYFYSSFSGTLPWASC

Dr_SLC6A4A  213 NNRWNTPNCTHYLSTDLNVSWTNNSISPAEEFYVRQVLQVHLSPGLHQLGWVSWQLALCL
Ol_SLC6A4A  175 TNSWNTVNCYRYLSSDQNVTWSNLSTSPAEEFYTRQVLQVHLSSGLHQLGSVSWQLALCL
GG_SLC6A4A  241 NNAWNTGNCTTYFSKD-NISWALHSISPAEEFYTRQVLQVHRSNGLDDLGGISWQLTLCL
Hs_SLC6A4A  201 KNSWNTGNCTNYFSED-NITWTLHSTSPAEEFYTRHVLQIHRSKGLQDLGGISWQLALCI
Dr_SLC6A4B  160 DNDWNTENCTNYFGKD-NVTWTNYSRSPAEEFYTRNVLAVHESSGLGNVGYIRWQLMLCL
Ol_SLC6A4B  154 DNAWNTPDCTNYFGMD-NVTWTNSSRSPAEEFYTRNVLEIHKSSGLKNVGGVRWQLLLCL
Gg_SLC6A4B  121 DNPWNTPNCTNYFGRN-NVTWTNFSRSPAEEFYT--------------------------

Dr_SLC6A4A  273 LFIFTVVYFSIWKGVKTSGKVVWVTATFPYLVLLILLIRGATLPGAWRGVVFYLKPDWKK
Ol_SLC6A4A  235 LFIFTIVYFSIWKGVKTSGKVVWVTATFPYLVLLILLIRGATLPGAWRGVVFYLKPDWQK
GG_SLC6A4A  300 LLIFIIVYFSIWKGVKTSGKVVWVTATFPYVILFILLVRGATLPGAWRGVLYYLKPEWQK
Hs_SLC6A4A  260 MLIFTVIYFSIWKGVKTSGKVVWVTATFPYIILSVLLVRGATLPGAWRGVLFYLKPNWQK
Dr_SLC6A4B  219 FLIFTIVYFSLWKGVKTSGKVVWVTATLPYVVLLILMIRGATLPGAWKGVVFYLNPKWEK
Ol_SLC6A4B  213 FLIFTIVYFSLWKGVKTSGKVVWVTATLPYIVLFILLIRGATLPGAWRGVVFYLKPQWEK
Gg_SLC6A4B  154 -----IVYFSLWKGVKTSGKVVWVTATLPYLVLLILLLRGATLPGAWRGVLFYLRPDWGK

Dr_SLC6A4A  333 LLTTTVWLDAAAQIFFSLGPGFGVLLAFASYNPFHNNCYKDALITSSVNCLTSFLSGFVI
Ol_SLC6A4A  295 LLSTTVWIDAAAQIFFSLGPGFGVLLAFASYNPFHNNCYKDALVTSSVNCLTSFLSGFVI
GG_SLC6A4A  360 LLATEVWVDAAAQIFFSLGPGFGVLLAYASYNKFHNNCYQDALVTSTVNCLTSFVSGFVI
Hs_SLC6A4A  320 LLETGVWIDAAAQIFFSLGPGFGVLLAFASYNKFNNNCYQDALVTSVVNCMTSFVSGFVI
Dr_SLC6A4B  279 LKETSVWVDAAAQIFFSLGPGFGVLLALSSYNPFTNNCYRDAIVTSLVNCLTSFVSGFVI
Ol_SLC6A4B  273 LLETSVWVDAAAQIFFSLGPGFGVLLALSSYNPFTNNCYRDAIVTSLVNCLTSFVSGFVI
Gg_SLC6A4B  209 LLSTAVWVDAAAQIFFSLGPGFGVLLALASYNHFHNNCYRDALITSAVNCLTSFLSGFVI

Dr_SLC6A4A  393 FTVLGYMAEMRQQGVETVAKDAGPSLLFIIYAEAIANMPAATFFAIIFFLMIIMLGLDST
Ol_SLC6A4A  355 FTVLGYMAEMRQQNVDVVANHPGPSLLFIIYAEAIANMPAATFFAIIFFLMIIMLGLDST
GG_SLC6A4A  420 FTVLGYMAEMRNEDVSEVAKDMGPSLLFITYAEAIANMPASTFFAIIFFLMLLTLGLDST
Hs_SLC6A4A  380 FTVLGYMAEMRNEDVSEVAKDAGPSLLFITYAEAIANMPASTFFAIIFFLMLITLGLDST
Dr_SLC6A4B  339 FTVLGYMAEQRNVNVEDVARDKGPSLLFITYPEAIANMVGSTFFAIIFFVMMITLGLDST
Ol_SLC6A4B  333 FTVLGYMAEMRKVEVEDVAKDKGPSLLFITYPEAIANMMGSTFFAIIFFVMMISLGLDST
Gg_SLC6A4B  269 FTVLGYMAEMRDVEVEDVARDKGPSLLFITYPEAIANMVGSTFFAIIFFLMMITLGLDST

Dr_SLC6A4A  453 FAGLEGVITAMLDEFPHLLARRREWFVLGLVCVCYLGALSTLTYGGAFVVKLFEEYATGP
Ol_SLC6A4A  415 FAGLEGVITAMLDEFPRLLAKRREWFVFGLVCVCYLGALSTLTYGGAFVVKLFEEYATGP
GG_SLC6A4A  480 FAGLEGVITGVLDEFPHVWSKRREFFVLGLIIICFLGSLATLTFGGAYVVKLFEEYATGP
Hs_SLC6A4A  440 FAGLEGVITAVLDEFPHVWAKRRERFVLAVVITCFFGSLVTLTFGGAYVVKLLEEYATGP
Dr_SLC6A4B  399 FGGLEAIITAVMDEYPDVLSHRRELFVLGLVVVCFLGSLSTLTKGGAYVVKLLEEFGVGS
Ol_SLC6A4B  393 FGGLEAIITAVLDEYPDQLSHRRELFVLCLVVVCFLGSLSTLTYGGAYVVKLLEEFGVGC
Gg_SLC6A4B  329 FGGLEAVITAVMDEYPQVLARHRELFVLGLITVCFLGSLSTLT-GGAYVVKLLEEFGAGC

Dr_SLC6A4A  513 AVITVVFLEVIAVSWFYGTTRFCNDVQLMLGFAPGLFWRVCWIAICPCFLLFIIVSFLAF
Ol_SLC6A4A  475 AVITVVLLEVIAVSWFYGTSRFCNDIQVMLGFYPGCFWRVCWVAICPCFLLFIIISFLAF
GG_SLC6A4A  540 AVLTVVFLEAVAVAWFYGITQFCNDVKEMLGFAPGWYWRVCWVAISPIFLLFVTCSFLSN
Hs_SLC6A4A  500 AVLTVALIEAVAVSWFYGITQFCRDVKEMLGFSPGWFWRICWVAISPLFLLFIICSFLMS
Dr_SLC6A4B  459 SIIAIVFLEATAVSWFYGINRFSNDIKSMLGYTPGLFWKVCWVAISPAFLAYIIISSLLN
Ol_SLC6A4B  453 SIIAVGFLEAIAVSWFYGINRFSNDVQAMLGKAPGLFWRVCWVGISPAFLAYIIVSSLLK
Gg_SLC6A4B  388 SILAVVLLETIAVSWFYGIQRFSHDVKAMLGFTPGLFWKVCWV------------SSLLE

Dr_SLC6A4A  573 PPEVKLFDYLYPFWTTVLGYCIGVSSFICVPSYMVYHLVTTKGTFQQRLLKGITPEAPGS
Ol_SLC6A4A  535 PPEVKLFNYTYPPWTTVLGYCIGVSSFICVPSYMVYYLLNAKGTFKQRLLKSITPEPSSE
GG_SLC6A4A  600 PPELRLFDYNYPYWTTVVGYCIGTSSIICIPIYMAYRLIITPGTLKERILKSITPETATE
Hs_SLC6A4A  560 PPQLRLFQYNYPYWSIILGYCIGTSSFICIPTYIAYRLIITPGTFKERIIKSITPETPTE
Dr_SLC6A4B  519 PQTLTLFDYEFPDWSITVGYIIGASSFIWIPIYMVYKLVWTPGSLKQRLAVCLRPERTLP
Ol_SLC6A4B  513 APPLTLFDYKYPDWSITVGYIIGFSSFMWIPIYMVYKLVWTPGSLKQRLAVCLRPER---
Gg_SLC6A4B  436 QPPLALFGYQYPAWSTSLGHLIGASSFICIPVYMVYKLVWTPGSLKQRLAVCIRPEKT--

Dr_SLC6A4A  633 SGPQRDTIVITNAV
Ol_SLC6A4A  595 NH------------
GG_SLC6A4A  660 IPFGDIRMNAV---
Hs_SLC6A4A  620 IPCGDIRLNAV---
Dr_SLC6A4B  579 DIHTDGMSLSPVP-
Ol_SLC6A4B      --------------
Gg_SLC6A4B      --------------
```

Supplementary Figure 5

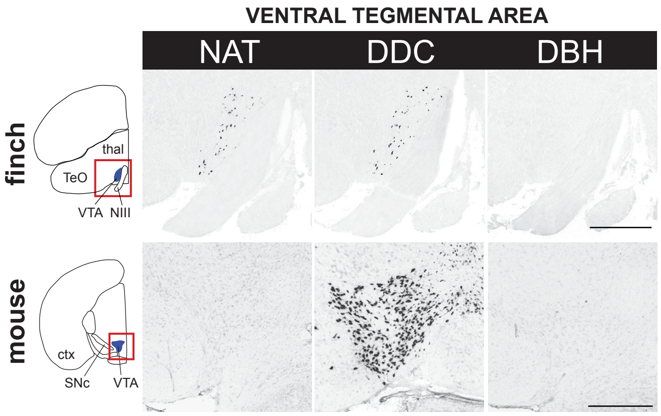

Supplementary Figure 6

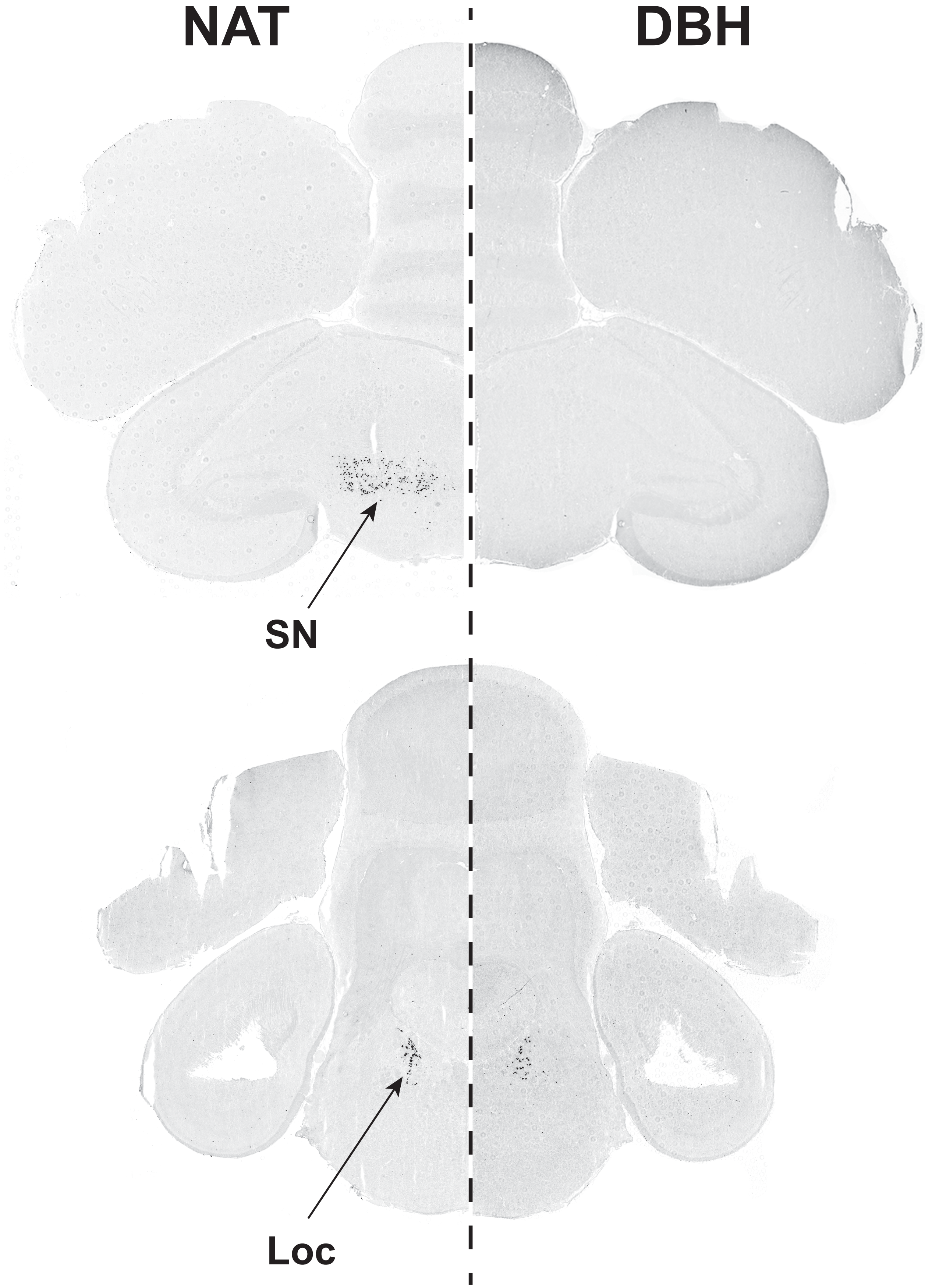

Supplementary Figure 7

```
chicken NAT   1 MGQRHPQCYLARMNKQIQPDRKVSVVAPLPERPGPSPPRKDVELILVKEHNGVQYTSSNLLPAAPAPRYG
  finch NAT   1 ~~~~~~~MFKARMNKQVQPERKVSVVAPLPDRAGPPPPPKKDVELILVK----EHNGVHSLLPAAPAPRG
  mouse NAT   1 ~~~~~~~~~~~~~~~~~~MLLARMNPQVQPELGGADPLPEQPLRPCKTADLLVVKERNGVQCLLASQDSD
  human NAT   1 ~~~~~~~~~~~~~~~~~~MLLARMNPQVQPENNGADTGPEQPLRARKTAELLVVKERNGVQCLLAPRDGD

  human DAT   1 ~~~~~~~~~~~~~~MSKSKCSVGLMSSVVAPAKEPNAVGPKEVELILVKEQNGVQLTSSTLTNPRQSPVE

                TM1  TM2
chicken NAT  71 PQDRETWGKKIDFLLSVIGFAVDLANVWRFPYLCYKNGGGAFLIPYILFLIIAGMPLFYMELALGQYNRE
  finch NAT  60 TQDRETWGKKIDFLLSVIGFAVDLANVWRFPYLCYKNGGGAFLIPYILFLIIAGMPLFYMELALGQYNRE
  mouse NAT  53 AQPRETWGKKIDFLLSVVGFAVDLANVWRFPYLCYKNGGGAFLIPYTLFLIIAGMPLFYMELALGQYNRE
  human NAT  53 AQPRETWGKKIDFLLSVVGFAVDLANVWRFPYLCYKNGGGAFLIPYTLFLIIAGMPLFYMELALGQYNRE
                                                                           *
  human DAT  57 AQDRETWGKKIDFLLSVIGFAVDLANVWRFPYLCYKNGGGAFLVPYLLFMVIAGMPLFYMELALGQFNRE

                TM3
chicken NAT 141 GAATVWKICPVFKGVGYAVILIALYVGFYYNVIIAWSLYYLFSSFTFELPWTNCDNSWNSPNCTDPKLFN
  finch NAT 130 GAATVWKICPVFKGVGYAVILIALYVGFYYNVIIAWSLYYLFSSFTFELPWTNCHNSWNSPNCTDPKLFN
  mouse NAT 123 GAATVWKICPFFKGVGYAVILIALYVGFYYNVIIAWSLYYLFASFTLNLPWTNCGHSWNSPNCTDPKLLN
  human NAT 123 GAATVWKICPFFKGVGYAVILIALYVGFYYNVIIAWSLYYLFSSFTLNLPWTDCGHTWNSPNCTDPKLLN

  human DAT 127 GAAGVWKICPILKGVGFTVILISLYVGFFYNVIIAWALHYLFSSFTTELPWIHCNNSWNSPNCSDAHPGD

                TM4
chicken NAT 211 ASVLGNGTKYSKYKLTPAAEFYERGVLHLHESRGIHDLGLPRWQLSLCLLVVVIILFFSLWKGVKTSGKV
  finch NAT 200 ASVLGNGTKYSKYKLTPAAEFYERGVLHLHESRGIHDLGLPRWQLALCLLVVVIILFFSLWKGVKTSGKV
  mouse NAT 193 ASVLGDHTKYSKYKFTPAAEFYERGVLHLHESSGIHDIGLPQWQLLLCLMVVIVVLYFSLWKGVKTSGKV
  human NAT 193 GSVLGNHTKYSKYKFTPAAEFYERGVLHLHESSGIHDIGLPQWQLLLCLMVVIVVLYFSLWKGVKTSGKV

  human DAT 197 SSGDSSGLN-DTFGTTPAAEYFERGVLHLHQSHGIDDLGPPRWQLTACLVLVIVLLYFSLWKGVKTSGKV

                TM5  TM6
chicken NAT 281 VWITATLPYVVLFVLLIHGITLPGAYNGINAYLHIDFRRLKEATVWIDAATQIFYSLGAGFGVLIAFASY
  finch NAT 270 VWITATLPYVVLFVLLIHGITLPGAYNGINAYLYIDFRRLKEATVWIDAATQIFYSLGAGFGVLIAFASY
  mouse NAT 263 VWITATLPYFVLFVLLVHGVTLPGASNGINAYLHIDFYRLKEATVWIDAATQIFFSLGAGFGVLIAFASY
  human NAT 263 VWITATLPYFVLFVLLVHGVTLPGASNGINAYLHIDFYRLKEATVWIDAATQIFFSLGAGFGVLIAFASY

  human DAT 265 VWITATMPYVVLTALLLRGVTLPGAIDGIRAYLSVDFYRLCEASVWIDAATQVCFSLGVGFGVLIAFSSY

                TM7
chicken NAT 351 NKFDNNCYRDALLTSTINCVTSFISGFAIFSILGYMAHEHKVKIEDVATEGAGLVFILYPEAISTLSGST
  finch NAT 340 NKFDNNCYRDALLTSTINCVTSFISGFAIFSILGYMAHEHKVKIEDVATEGAGLVFILYPEAISTLSGST
  mouse NAT 333 NKFDNNCYRDALLTSTINCVTSFISGFAIFSILGYMAHEHKVNIEDVATEGAGLVFILYPEAISTLSGST
  human NAT 333 NKFDNNCYRDALLTSSINCITSFISGFAIFSILGYMAHEHKVNIEDVATEGAGLVFILYPEAISTLSGST

  human DAT 335 NKFTNNCYRDAIVTTSINSLTSFSSGFVVFSFLGYMAQKHSVPIGDVAKDGPGLIFIIYPEAIATLPLSS

                TM8  TM9
chicken NAT 421 FWAVVFFIMLLTLGIDSSMGGMEAVITGLADDFHILKQHRKLFTFGVSFGTFLLALFCITNGGIYVLTLL
  finch NAT 410 FWAVVFFIMLLTLGIDSSMGGMEAVITGLADDFHLLKQHRKLFTFGVSFGTFLLGLFCITNGGIYVLTLL
  mouse NAT 403 FWAVLFFLMLLALGLDSSMGGMEAVITGLADDFQVLKRHRKLFTCVVTISTFLLALFCITKGGIYVLTLL
  human NAT 403 FWAVVFFVMLLALGLDSSMGGMEAVITGLADDFQVLKRHRKLFTFGVTFSTFLLALFCITKGGIYVLTLL

  human DAT 405 AWAVVFFIMLLTLGIDSAMGGMESVITGLIDEFQLLHRHRELFTLFIVLATFLLSLFCVTNGGIYVFTLL

                TM10  TM11
chicken NAT 491 DTFAAGTSILFAVLMEAIGVSWFYGVDRFSEDIQQMMGFKPGLYWRLCWKFVSPAFLLFVVIVSIINFKP
  finch NAT 480 DTFAAGTSILFAVLMEAIGVSWFYGVDRFSEDIQQMMGFKPGLYWRLCWKFVSPAFLLFVVIVSIINFKP
  mouse NAT 473 DTFAAGTSILFAVLMEAIGVSWFYGVDRFSNDIQQMMGFKPGLYWRLCWKFVSPAFLLFVVVVSIINFKP
  human NAT 473 DTFAAGTSILFAVLMEAIGVSWFYGVDRFSNDIQQMMGFRPGLYWRLCWKFVSPAFLLFVVVVSIINFKP
                *
  human DAT 475 DHFAAGTSILFGVLIEAIGVAWFYGVGQFSDDIQQMTGQRPSLYWRLCWKLVSPCFLLFVVVVSIVTFRP

                TM12
chicken NAT 561 LTYDDYTFPPWANRIGWGIALSSMILVPAYVIYKFMNVRGTFKERLAYCITPENEHQLVAQGNVRQFKLQ
  finch NAT 550 LTYDDYTFPPWANRIGWGIALSSMILVPAYIIYKFMNVRGTFKERLAYCITPENEHQLVAQGNVRQFKLQ
  mouse NAT 543 LTYDDYTYPPWANWVGWGIALSSMILVPAYVIYKFLSIRGSLWERVAYGITPENEHHLVAQRDVRQFQLR
  human NAT 543 LTYDDYIFPPWANWVGWGIALSSMVLVPIYVIYKFLSTQGSLWERLAYGITPENEHHLVAQRDIRQFQLQ

  human DAT 545 PHYGAYIFPDWANALGWVIATSSMAMVPIYAAYKFCSLPGSFREKLAYAIAPEKDRELVDRGEVRQFTLR

chicken NAT 631 HWLTI
  finch NAT 620 HWLAI
  mouse NAT 613 HWLAI
  human NAT 613 HWLAI
  human DAT 615 HWLKV
```

Supplementary Figure 8

		Medaka (NAT)	Zebrafish (NAT)	Anole (NAT)	Chicken (NAT)	Z.finch (NAT)	Mouse (NAT)	Human (NAT)	Medaka (DAT)	Zebrafish (DAT)	Mouse (DAT)	Human (DAT)
**NAT**	Medaka	100.0%										
	Zebrafish	79.0%	100.0%									
	Anole	66.1%	60.4%	100.0%								
	Chicken	66.9%	63.0%	77.7%	100.0%							
	Z. finch	67.3%	63.9%	78.3%	92.0%	100.0%						
	Mouse	64.9%	62.4%	73.8%	80.3%	81.2%	100.0%					
	Human	65.2%	62.6%	73.7%	80.0%	81.0%	94.6%	100.0%				
**DAT**	Medaka	64.6%	61.6%	56.9%	60.2%	59.8%	58.6%	58.9%	100.0%			
	Zebrafish	59.0%	55.0%	60.7%	64.6%	63.5%	63.2%	63.5%	76.7%	100.0%		
	Mouse	58.4%	55.8%	61.0%	65.2%	64.3%	64.6%	65.5%	70.4%	76.3%	100.0%	
	Human	58.6%	56.2%	61.3%	65.9%	64.9%	65.3%	65.9%	71.0%	77.0%	93.8%	100%
